# Safety and Pharmacokinetics of Islatravir in Individuals with Severe Renal Insufficiency

**DOI:** 10.1128/aac.00931-22

**Published:** 2022-11-08

**Authors:** Randolph P. Matthews, Youfang Cao, Munjal Patel, Vanessa L. Weissler, Arinjita Bhattacharyya, Inge De Lepeleire, Stefanie Last, Juan C. Rondon, Ryan Vargo, S. Aubrey Stoch, Marian Iwamoto

**Affiliations:** a Merck & Co., Inc., Rahway, New Jersey, USA; b MSD (Europe) Inc., Brussels, Belgium; c Charité Research Organization, Berlin, Germany; d Clinical Pharmacology of Miami, Miami, Florida, USA

**Keywords:** chronic kidney disease, human immunodeficiency virus, phase 1, renal impairment, reverse transcriptase translocation inhibitor

## Abstract

Islatravir (MK-8591) is a high-potency reverse transcriptase translocation inhibitor in development for the treatment of HIV-1 infection. Data from preclinical and clinical studies suggest that ~30% to 60% of islatravir is excreted renally and that islatravir is not a substrate of renal transporters. To assess the impact of renal impairment on the pharmacokinetics of islatravir, an open-label phase 1 trial was conducted with individuals with severe renal insufficiency (RI). A single dose of islatravir 60 mg was administered orally to individuals with severe RI (estimated glomerular filtration rate [eGFR] <30 mL/min/1.73 m^2^) and to healthy individuals without renal impairment (matched control group; eGFR ≥90 mL/min/1.73 m^2^). Safety and tolerability were assessed, and blood samples were collected to measure the pharmacokinetics of islatravir and its major metabolite 4’-ethynyl-2-fluoro-2’deoxyinosine (M4) in plasma, as well as active islatravir-triphosphate (TP) in peripheral blood mononuclear cells (PBMCs). Plasma islatravir and M4 area under the concentration-time curve from zero to infinity (AUC_0-∞_) were ~2-fold and ~5-fold higher, respectively, in participants with severe RI relative to controls, whereas islatravir-TP AUC_0-∞_ was ~1.5-fold higher in the RI group than in the control group. The half-lives of islatravir in plasma and islatravir-TP in PBMCs were longer in participants with severe RI than in controls. These findings are consistent with renal excretion playing a major role in islatravir elimination. A single oral dose of islatravir 60 mg was generally well tolerated. These data provide guidance regarding administration of islatravir in individuals with impaired renal function. (This study has been registered at ClinicalTrials.gov under registration no. NCT04303156.)

## INTRODUCTION

Islatravir (MK-8591) is an investigational deoxyadenosine analog that suppresses HIV replication via reverse transcriptase translocation inhibition. The novel structure of islatravir confers potent activity and a high barrier to the selection of drug-resistant HIV variants (1–6). Islatravir demonstrated robust antiviral efficacy against wild-type and common drug-resistant variants of HIV-1 *in vitro* and in preclinical animal models of HIV-1 infection (4, 7–10). Supported by its potency, pharmacokinetics (PK), and drug-drug interaction profile (6, 11, 12), daily and weekly islatravir dosing regimens are in development for the treatment of HIV-1, and a once-monthly oral regimen and a once-yearly implant regimen are in development for the prevention of HIV-1 (13–15). In a phase 2b clinical trial in treatment-naive adults with HIV-1, once-daily oral islatravir plus doravirine demonstrated durable efficacy through 96 weeks; 81.1% of participants treated with islatravir plus doravirine and 80.6% of participants in the comparator group maintained HIV-1 RNA <50 copies/mL, with low incidence of protocol-defined virologic failure through 96 weeks in both groups (16).

After single-dose oral administration, islatravir is rapidly absorbed, with a median plasma time of maximum concentration (T_max_) of 0.5 h and an apparent terminal half-life (t_½_) of 49 h to 61 h (6). Islatravir is converted intracellularly to its active form islatravir-triphosphate (islatravir-TP) (5); the apparent terminal plasma t_½_ for islatravir-TP is 177 h to 209 h, which allows for extended-duration dosing (12). Islatravir displays dose-proportional PK over a wide range of doses (0.25 mg to 400 mg) (6, 12). Islatravir is eliminated by renal excretion of the unchanged parent drug and adenosine deaminase–mediated metabolism to the major circulating islatravir metabolite 4’-ethynyl-2-fluoro-2’deoxyinosine (M4) (11). Approximately 30% to 60% of plasma islatravir is excreted renally. Results of preclinical studies suggest that islatravir is not a substrate of renal transporters (11, 17); therefore, renal excretion of islatravir seems largely mediated via glomerular filtration.

As HIV-1 treatments extend life expectancy, impaired renal function is becoming an increasingly prevalent comorbidity in people living with HIV-1 (18). People living with HIV-1 are at higher risk than the general population of acute kidney injury, HIV-associated kidney disease, comorbid chronic kidney disease, and treatment-related kidney toxic effects (18). Factors that contribute to the higher risk of kidney disease include advancing age, cardiometabolic risk factors, adverse effects of combination antiretroviral therapy, and traditional risk factors such as obesity and hypertension (19–22). Understanding the effects of renal impairment on the safety and PK of islatravir is important because renal insufficiency (RI) may alter plasma levels of islatravir or intracellular levels of islatravir-TP. To assess the impact of RI on islatravir PK, a clinical study (ClinicalTrials.gov: NCT04303156) was designed to evaluate the PK, safety, and tolerability of a single oral dose of islatravir 60 mg in participants with severe RI compared with matched healthy controls.

## RESULTS

### Participants.

A total of six participants with severe RI and six healthy matched control participants with adequate renal function were enrolled. Participant demographics and baseline characteristics are summarized in Table 1. Baseline demographics were generally balanced between the two groups, with the exception of renal function and comorbid conditions. Comorbid conditions in the severe RI group (number of participants with condition) included the following: hypertension (six), hyperlipidemia/hypercholesterolemia (five), type 2 diabetes mellitus (four), coronary artery disease (two), hyperuricemia/gout (three), vitamin D deficiency (two), anemia (one), and hypothyroidism (one). Concomitant medications were allowed for participants with severe RI and are listed in the online supplemental materials.

**TABLE 1:** Participant demographics and baseline characteristics^*a*^

Parameter	Severe RI (*n *= 6)	Healthy matched control (*n *= 6)
Female, *n*	1	2
Age, mean (SD), y	57.7 (12.9)	58.8 (5.5)
Range	35 to 73	53 to 66
Race, *n*		
Black or African American	0	1
White	6	5
Ethnicity, *n*		
Hispanic or Latino	3	3
Not Hispanic or Latino	3	3
Weight, mean (SD), kg	83.2 (14.7)	80.9 (4.9)
BMI, mean (SD), kg/m^2^	29.5 (4.1)	27.5 (2.7)
Range	25 to 37	24 to 32
eGFR,^*b*^ mean (SD), mL/min/1.73 m^2^	21.2 (6.2)	97.2 (5.4)

a BMI, body mass index; eGFR, estimated glomerular filtration rate; RI, renal insufficiency.

b Baseline eGFR based on the Chronic Kidney Disease Epidemiology Collaboration was obtained twice (≥72 h apart) during participant screening, and mean values were used to determine renal status.

### Plasma islatravir and M4 PK.

**FIG 1:**
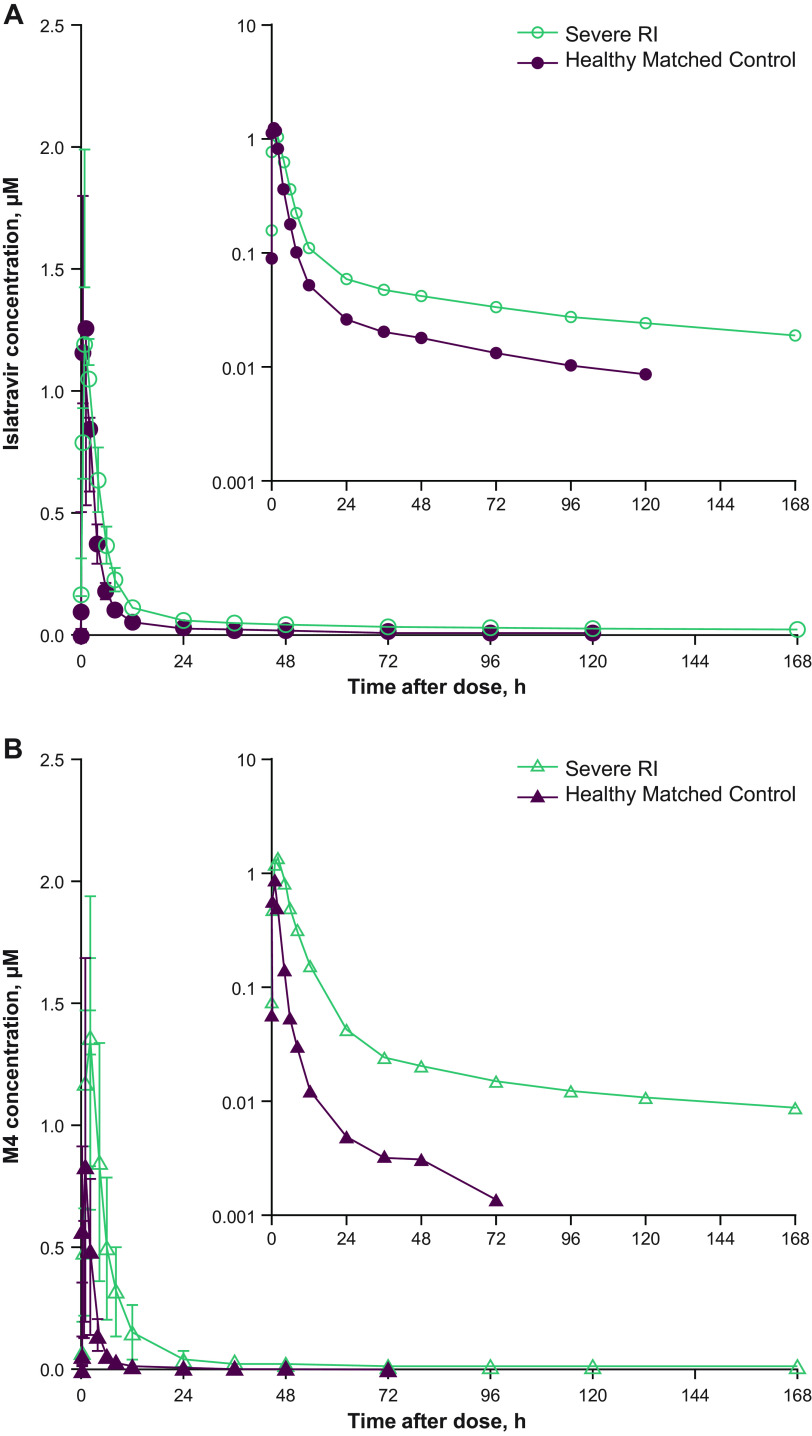
Plasma concentration versus time profiles of islatravir and M4. Arithmetic mean (± SD) plasma concentration versus time profiles of (A) islatravir and (B) M4 after administration of a single oral dose of islatravir 60 mg to participants with severe RI and matched healthy control participants (*n *= 6 per condition); inset: semi-log plot. M4, 4’-ethynyl-2-fluoro-2’deoxyinosine; RI, renal insufficiency.

The mean islatravir and M4 plasma concentration-time profiles are shown in Fig. 1, and PK parameter values are shown in Table 2. After administration of a single dose of islatravir 60 mg orally, geometric mean ratio (90% CI) of the area under the concentration-time curve from zero to infinity (AUC_0-∞_) of plasma islatravir and of plasma M4 was 2.20-fold (1.68 to 2.88) and 5.28-fold (3.27 to 8.53) higher, respectively, in participants with severe RI than in healthy controls. In contrast, the maximum plasma concentration (C_max_) of plasma islatravir was similar, whereas the C_max_ of plasma M4 was ~2-fold higher in participants with severe RI than in healthy control participants. The apparent terminal t_1/2_ of plasma islatravir and of M4 was prolonged, and apparent clearance after extravascular administration (CL/F) was lower in participants with severe RI than in healthy control participants; apparent volume of distribution during the terminal phase (Vz/F) was comparable between the two groups.

**TABLE 2:** Summary of plasma islatravir and plasma M4 PK in participants with severe RI and healthy matched control participants after administration of a single dose of islatravir 60 mg^*a*^

Analyte (matrix)	PK parameter	GM (95% CI)		GMR (90% CI)
		Severe RI (*n *= 6)	Healthy matched control (*n *= 6)	Severe RI/healthy matched control
Islatravir (plasma)	AUC_0-∞_,^*b*^ h·μM	14.4 (11.8 to 17.6)	6.54 (4.77 to 8.98)	2.20 (1.68 to 2.88)
	AUC_0-last_,^*b*^ h·μM	11.0 (9.17 to 13.1)	5.68 (4.06 to 7.94)	1.93 (1.46 to 2.55)
	C_max_,^*b*^ μM	1.23 (1.06 to 1.42)	1.19 (0.699 to 2.04)	1.03 (0.67 to 1.57)
	T_max,_ median (min, max), h	1.03 (1.00, 2.00)	0.75 (0.50, 1.00)	NA
	t_1/2_,^*c*^ h	127 (7.7)	72.0 (15.5)	NA
	CL/F,^*b*^ L/h	14.2 (11.6 to 17.4)	31.3 (22.8 to 42.9)	0.46 (0.35 to 0.60)
	Vz/F,^*b*^ L	2,610 (2,170 to 3,140)	3,250 (2,100 to 5,030)	0.80 (0.56 to 1.14)
M4 (plasma)	AUC_0-∞_,^*b*^ h·μM	10.7 (6.97 to 16.6)	2.04 (1.21 to 3.43)	5.28 (3.27 to 8.53)
	AUC_0-last_,^*b*^ h·μM	9.36 (6.13 to 14.3)	1.97 (1.17 to 3.32)	4.74 (2.95 to 7.62)
	C_max_,^*b*^ μM	1.34 (0.935 to 1.93)	0.737 (0.338 to 1.61)	1.82 (0.97 to 3.43)
	T_max_ median (min, max), h	2.00 (1.00, 2.03)	1.00 (0.50, 1.95)	NA
	t_1/2_,^*c*^ h	125 (27.8)	22.7 (79.3)	NA
	CL/F,^*b*^ L/h	19.0 (12.3 to 29.2)	100 (59.4 to 169)	0.19 (0.12 to 0.31)
	Vz/F,^*b*^ L	3,420 (2,070 to 5,650)	3,280 (2,190 to 4,920)	1.04 (0.66 to 1.64)

a AUC_0-last_, area under the concentration-time curve from time 0 to last sampling time after dose; AUC_0-∞_, area under the concentration-time curve from before dose to infinity; CL/F, apparent clearance after extravascular administration; C_max_, maximum measured concentration; GCV, geometric coefficient of variation; GM, geometric mean; GMR, geometric mean ratio; M4, 4’-ethynyl-2-fluoro-2’deoxyinosine; NA, not applicable; PK, pharmacokinetics; RI, renal insufficiency; t_1/2_, apparent terminal half-life; T_max_, time to maximum measured concentration; Vz/F, apparent volume of distribution during the terminal phase.

b Back-transformed least squares mean and 95% CI from fixed-effects model performed on natural log-transformed values.

c Geometric mean (%GCV).

### Peripheral blood mononuclear cells islatravir-TP PK.

**FIG 2:**
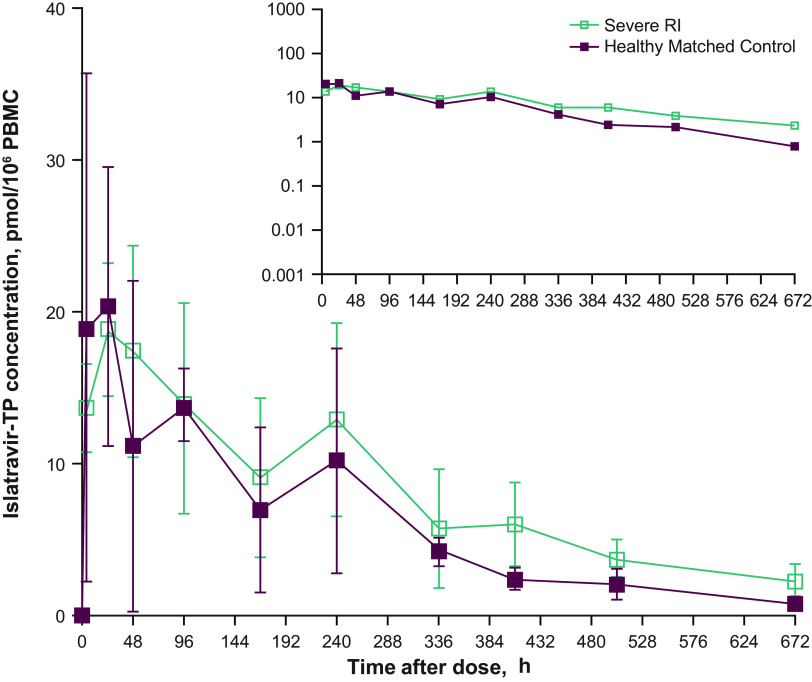
PBMC concentration versus time profiles of islatravir-TP. Arithmetic mean (± SD) PBMC concentration versus time profiles of islatravir-TP after administration of a single oral dose of islatravir 60 mg to participants with severe RI and matched healthy control participants (*n *= 6 per condition); inset: semi-log plot. Islatravir-TP, islatravir-triphosphate; PBMC, peripheral blood mononuclear cells; RI, renal insufficiency.

Fig. 2 shows the mean islatravir-TP intracellular concentration-time profile, and Table 3 shows the PK parameter values. AUC_0-∞_ of intracellular islatravir-TP was 1.5-fold higher in participants with severe RI than in healthy control participants. The C_max_ of intracellular islatravir-TP was similar between groups. At 168 h and 672 h after dosing, the concentrations of intracellular islatravir-TP were higher in participants with severe RI than in healthy control participants. The calculated ratio of peripheral blood mononuclear cells (PBMCs) islatravir-TP to plasma islatravir AUC_0-∞_ in μM*hour, based on the results shown in Tables 2 and 3, is 2,017 for the severe RI participants and 2,997 for the matched control group.

**TABLE 3:** Summary of PBMC islatravir-TP PK in participants with severe RI and healthy matched control participants after administration of a single dose of islatravir 60 mg^*a*^

PK parameter	GM (95% CI)		GMR (90% CI)
	Severe RI (*n *= 6)	Healthy matched control (*n *= 6)	Severe RI/healthy matched control
AUC_0-∞,_^*b*^ h·pmol/10^6^ PBMCs	5,810 (3,890 to 8,700)	3,920 (2,830 to 5,420)	1.48 (1.03 to 2.14)
AUC_0-last_,^*b*^ h·μM/10^6^ PBMCs	5,200 (3,670 to 7,370)	3,780 (2,720 to 5,230)	1.38 (0.98 to 1.93)
C_max_,^*b*^ pmol/10^6^ PBMCs	20.3 (15.1 to 27.4)	21.6 (13.7 to 34.1)	0.94 (0.64 to 1.39)
C_24_,^*b*^ pmol/10^6^ PBMCs	18.4 (14.2 to 23.8)	19.0 (12.9 to 28.2)	0.97 (0.69 to 1.35)
C_168_,^*b*^ pmol/10^6^ PBMCs	8.06 (4.69 to 13.9)	4.43 (0.938 to 20.9)^*c*^	1.82 (0.55 to 6.02)
C_672_,*^b^* pmol/10^6^ PBMCs	1.96 (0.982 to 3.92)	0.730 (0.489 to 1.09)	2.69 (1.51 to 4.80)
T_max_, median (min, max), h	36.00 (24.00, 240.12)	24.00 (4.00, 239.78)	NA
t_1/2_,^*d*^ h	181 (48.4)	131 (19.0)	NA

a AUC_0-last_, area under the concentration-time curve from time zero to last sampling time after dose; AUC_0-∞_, area under the concentration-time curve from before dose to infinity; C, concentration at hours after dosing; C_max_, maximum measured concentration; GCV, geometric coefficient of variation; GM, geometric mean; GMR, geometric mean ratio; islatravir-TP, islatravir-triphosphate; NA, not applicable; PBMC, peripheral blood mononuclear cells; PK, pharmacokinetics; RI, renal insufficiency; t_1/2_, apparent terminal half-life; T_max_, time to maximum measured concentration.

b Back-transformed least squares mean and 95% CI from fixed-effects model performed on natural log-transformed values.

c *n *= 5, 1 participant had missing sample at 168-h nominal time point.

d Geometric mean (%GCV).

### Safety.

A single oral dose of islatravir 60 mg had a favorable safety profile in both groups of participants. Only one participant (16.7%) in the severe RI group experienced an adverse event (AE; extremity pain), which was not considered drug related. No serious AEs were reported, and no participants discontinued because of AEs. Although the study was conducted during the coronavirus disease 2019 (COVID-19) pandemic, no participants discontinued because of severe acute respiratory syndrome coronavirus 2 (SARS-CoV-2) or COVID-19 positivity. There were no significant findings with respect to vital signs, laboratory safety tests, or electrocardiogram results in either group.

## DISCUSSION

Islatravir is an HIV deoxyadenosine analog in development, and promising clinical efficacy data show its potential in the treatment and prevention of HIV (13, 16, 23, 24). To evaluate the PK and safety of islatravir, a phase 1 clinical study was conducted in individuals with severe RI.

Based on previous preclinical and clinical PK data, plasma islatravir and PBMC islatravir-TP levels were expected to increase in participants with severe RI relative to healthy control participants, considering islatravir undergoes renal clearance (6, 12, 13, 23, 25). The results of the current study suggest that plasma islatravir and M4 exposure were, in fact, higher in participants with severe RI, consistent with renal excretion as a relatively major factor in islatravir elimination (11). Renal impairment had a larger effect on M4 plasma exposure than on islatravir, which may be a result of greater reliance of M4 on renal excretion for elimination than islatravir. Although this trial has elucidated the effects of severe RI on the PK of islatravir, more modest effects are anticipated in individuals with mild or moderate RI than in those with severe RI.

Exposure of PBMC islatravir-TP was increased in participants with severe RI relative to those with normal renal function but to a lesser degree than exposure to plasma islatravir and M4. This apparent lower effect of severe RI on islatravir-TP exposure could simply be a delay in the effect on islatravir-TP because the initial concentrations of islatravir were generally similar between participants with severe RI and healthy matched controls. As islatravir concentrations persist owing to decreased clearance, islatravir-TP concentrations begin to build up as well. Islatravir-TP C_168_, the PK parameter associated with efficacy in the severe RI group, was higher than in the matched control group, supporting no expected decrease in efficacy (23, 26). The increase in islatravir-TP exposure is comparable with the range of previously studied dose levels of islatravir (6, 15, 24). Ongoing analysis of current trials will help elucidate whether the higher levels of islatravir-TP represent a potential safety risk (6).

The relationship between islatravir and islatravir-TP levels has been well characterized in healthy study participants and in participants living with HIV (6, 12, 23). The current study provides a greater understanding of that relationship in individuals with RI (14). The ratio of islatravir-TP to islatravir AUC_0-∞_ provides a gauge of the efficiency of phosphorylation, and, in previous studies, it was generally consistent after oral dosing (965:1 to 2,120:1) (6). In this study, similar ratios of islatravir-TP to islatravir AUC_0-∞_ of ~2,000:1 in participants with severe RI and ~3,000:1 in healthy control participants were observed. These ratios fall within the islatravir-TP assay variability and are not meaningfully different between severe RI and healthy control participants. The ratios in this study are therefore comparable with those observed previously in adults without HIV, suggesting that renal impairment does not significantly affect islatravir-TP PK distinct from the effect on islatravir parent PK.

In the current study, a single dose of islatravir 60 mg was generally well tolerated. Ongoing phase 2 and 3 studies, in which participants are exposed to islatravir for longer periods, will contribute to the understanding of more extensive exposure to islatravir and islatravir-TP (15, 24). Decreases in total lymphocyte and CD4^+^ T-cell counts have been observed in some participants receiving islatravir in phase 2 and phase 3 clinical studies (27). There was no meaningful change seen in lymphocyte counts in the present study; however, the present study has a very limited sample size with only a single dose of islatravir administered.

Limitations of the current study are small sample size, a relatively short exposure time to a single dose administration of islatravir, investigation of one dose level, and analysis of only severe RI. Because islatravir and islatravir-TP demonstrate dose-proportional behavior, data from the 60-mg dose are applicable across the therapeutic doses of interest across the clinical program. Based on the overall PK data of multiple dosing for islatravir, single-dose PK is predictive of multiple-dose behavior. However, time to equilibration of plasma islatravir and intracellular islatravir-TP may not be adequate to truly quantitate the effect of RI on islatravir-TP levels. As noted, data from individuals with severe RI can be extrapolated to those with mild and moderate RI. Individuals with end-stage renal disease and/or who are on dialysis were not included in the current study; therefore, no conclusions can be drawn regarding the PK and tolerability of islatravir in those individuals or regarding removal of islatravir by dialysis.

### Conclusion.

In the current study, severe RI affected the PK profile of a single oral dose of islatravir 60 mg, increasing islatravir plasma AUC on the order of ~2-fold, with a larger effect on the M4 metabolite AUC; intracellular islatravir-TP exposure was affected to a lesser degree. These data and additional data from ongoing islatravir trials will provide guidance regarding the need for dose adjustment of islatravir for individuals with impaired renal function.

## MATERIALS AND METHODS

This open-label phase 1 study (MK-8591-026) was conducted in accordance with the International Conference on Harmonization Good Clinical Practice Guidelines and the ethical principles set forth by the Declaration of Helsinki. The study and relevant supporting documents were approved by the IntegReview institutional review board (Austin, TX, USA) and the Landesamt für Gesundheit und Soziales ethics review committee (Berlin, Germany). All participants signed written informed consent before study entry. The study was conducted from June 2020 to October 2020.

### Participants.

Male and female participants aged 18 to 75 years with a body mass index of ≥18.5 and ≤40 kg/m^2^ were eligible for enrollment. For inclusion in the severe RI group, participants were required to have an estimated glomerular filtration rate (eGFR) of <30 mL/min/1.73 m^2^ based on the Chronic Kidney Disease Epidemiology Collaboration equation; participants undergoing dialysis were excluded. With the exception of RI, participants were to be in generally good health with stable chronic medical conditions. Rapidly fluctuating renal function (>30% difference between two measurements of eGFR taken ≥72 h apart) was exclusionary. Participants in the healthy matched control group were required to have an eGFR of ≥90 mL/min/1.73 m^2^. The healthy control group was matched by mean age (±10 years) and body mass (±10 kg) to the RI group. In addition, the number of healthy male and female participants was generally matched to the number of RI participants (within ±1 individual). Hepatitis B surface antigen, hepatitis C antibodies, and/or HIV positivity were exclusionary in both groups.

Concomitant medications to treat general medical conditions and/or conditions associated with renal disease were allowed in RI participants. Participants were required to be on a stable regimen for ≥1 month prior to islatravir administration and able to withhold the use within 4 h prior to and 8 h after islatravir administration. Drugs that might have interfered with the study were discontinued ≥2 weeks (or five half-lives of the compound, whichever was longer) prior to the first dosing of islatravir.

### Procedures.

All participants received a single oral dose of islatravir 60 mg (6 × 10 mg capsules) in the fasted state (participants fasted ≥8 h before dose administration and approximately 4 h after). A dose of 60 mg was selected, which is within a projected therapeutic dose range (28). The PK for both parent islatravir and islatravir-TP has been dose-proportional over the entire dose range evaluated to date (0.25 mg to 400 mg) (6), and thus, the effects of RI on PK can be extrapolated across doses.

Blood samples for plasma islatravir and M4 PK were drawn before dosing and at 0.25 h, 0.5 h, 1 h, 2 h, 4 h, 6 h, 8 h, 12 h, 24 h, 36 h, 48 h, 72 h, 96 h, and 120 h after dosing (healthy control group) or 0.25 h, 0.5 h, 1 h, 2 h, 4 h, 6 h, 8 h, 12 h, 24 h, 36 h, 48 h, 72 h, 96 h, 120 h, and 168 h after dosing (severe RI group). Blood samples to determine PBMC islatravir-TP PK were drawn after dosing and at 4 h, 24 h, 48 h, 96 h, 168 h, 240 h, 336 h, 408 h, 504 h, and 672 h after dosing for both groups. Safety was monitored throughout the study by repeated clinical and laboratory evaluations.

### PK assessments.

For plasma islatravir, plasma M4, and PBMC islatravir-TP analytes, liquid-liquid extraction, salt-assisted liquid-liquid extraction, and protein precipitation methods were used for isolation, respectively. After analyte isolation, liquid chromatography with tandem mass spectrometry was used for quantitation, as previously described (6, 11). The lower limit of quantitation was 0.02 ng/mL (0.0682 nM) for plasma islatravir, 0.5 ng/mL (1.70 nM) for plasma M4, and 0.1 ng/mL (0.188 nM) for PBMC islatravir-TP. PBMC cell counts (per 10^6^ cells) were estimated using a hemocytometer, and the conversion from nM to pmol/10^6^ cells was made using the standard assumption that 1 PBMC has an approximate volume of 0.2 pL (13, 29).

The PK parameters assessed for plasma islatravir and M4 were AUC_0-∞_, area under the concentration-time curve from zero to last sampling time after dose (AUC_0-last_), C_max_, T_max_, apparent terminal t_½_, CL/F, and Vz/F of plasma islatravir. The PK parameters assessed for islatravir-TP in PBMCs were AUC_0-∞_, AUC_0-last_, C_max_, concentration at 24 h after dosing (C_24_), C_168_, C_672_, T_max_, and apparent terminal t_½_. Plasma PK parameter values were calculated by noncompartmental analyses using Phoenix WinNonlin Professional software (version 8.1; Certara, Princeton, NJ, USA). AUC was calculated using the linear trapezoidal method for ascending concentrations and the log trapezoidal method for descending concentrations (linear-up/log-down).

### Safety assessments.

Safety and tolerability were assessed by evaluation of AEs, vital signs, electrocardiograms, and laboratory safety tests.

### Statistical analysis.

Individual values of each PK parameter and analyte were natural log-transformed and evaluated by use of a linear fixed-effects model containing a categorical effect for population (participants with severe RI, healthy participants). An unstructured covariance matrix was used to allow for unequal population variances via the REPEATED and GROUP statement in SAS PROC MIXED. The Kenward and Roger method was used to calculate the denominator degrees of freedom for the fixed effect. Ninety-five percent CIs for the least squares mean for each population were constructed on the natural log scale and referenced the t-distribution. Exponentiating the least squares mean and corresponding 95% CIs, yielded estimates for the population geometric mean and CIs about the geometric mean on the original scale. Percent geometric coefficient of variation (GCV) was calculated as, where s^2^ is the observed variance on the natural log scale. Results for participants with severe RI were compared with those for participants with normal renal function, using a two-sided 90% CI for the true difference in mean (participants with severe RI – healthy participants) for each PK parameter, using the mean square error from the model, and referenced a t-distribution. These confidence limits were exponentiated to obtain the 90% CI for the true ratio of geometric means (participants with severe RI/healthy participants) for each PK parameter.

### Data availability.

The data sharing policy, including restrictions, of Merck Sharp & Dohme LLC, a subsidiary of Merck & Co., Inc., Rahway, NJ, USA, is available at http://engagezone.msd.com/ds_documentation.php. Requests for access to the clinical study data can be submitted through the Engage Zone site or via email to dataaccess@merck.com.
