# Supplementary material for: Safety and Pharmacokinetics of Islatravir in Individuals with Severe Renal Insufficiency
Source: Antimicrob Agents Chemother. 2022 Nov 8;66(12):e00931-22. doi: 10.1128/aac.00931-22 (PMC9765080; doi:10.1128/aac.00931-22)
Supplement: Supplemental file 1 — Supplemental material. Download aac.00931-22-s0001.pdf, PDF file, 0.1 MB [file aac.00931-22-s0001.pdf]

Safety and Pharmacokinetics of Islatravir in Individuals with Severe Renal Insufficiency

Randolph P. Matthews,[a] Youfang Cao,[a] Munjal Patel,[a] Vanessa L. Weissler,[a] Arinjita Bhattacharyya,[a] Inge De Lepeleire,[b] Stefanie Last,[c] Juan C. Rondon,[d] Ryan Vargo,[a] S. Aubrey Stoch,[a] Marian Iwamoto[a]

[a]Merck & Co., Inc., Rahway, New Jersey, USA

[b]MSD (Europe) Inc., Brussels, Belgium

[c]Charité Research Organization, Berlin, Germany

[d]Clinical Pharmacology of Miami, Miami, Florida, USA

Supplemental Material

Listing of concomitant medications in participants with severe RI: acetylsalicylic acid, allopurinol, amlodipine, atorvastatin, bumetanide, candesartan, carvedilol, cholecalciferol, clonidine, clopidogrel, doxazosin, febuxostat, ferric maltol, folic acid, glipizide, hydrochlorothiazide, insulin glargine, insulin lispro, lercanidipine, levothyroxine, lisinopril, losartan, metformin, metoprolol, moxonidine, nebivolol, oxybutynin, paracetamol, ramipril, rosuvastatin, simvastatin, sodium bicarbonate, spironolactone, torsemide, and vitamin D National Osteoporosis Society (NOS).
